# *Schizochytrium limacinum* Supplementation in a Low Fish-Meal Diet Improved Immune Response and Intestinal Health of Juvenile *Penaeus monodon*

**DOI:** 10.3389/fphys.2020.00613

**Published:** 2020-06-30

**Authors:** Shiwei Xie, Dan Wei, Beiping Tan, Yongjian Liu, Lixia Tian, Jin Niu

**Affiliations:** ^1^Laboratory of Aquatic Animal Nutrition and Feed, Fisheries College, Guangdong Ocean University, Zhanjiang, China; ^2^Guangdong Provincial Key Laboratory of Improved Variety Reproduction in Aquatic Economic Animals, Institute of Aquatic Economic Animals, School of Life Sciences, Sun Yat-sen University, Guangzhou, China

**Keywords:** fish-meal, *Schizochytrium limacinum*, DHA, metabolomics, intestinal health

## Abstract

The aim of the present experiment was to evaluate the effects of *Schizochytrium limacinum* supplementation on the immune response, gut microbiota, and health of *Penaeus monodon* fed a low fish-meal (FM) diet. A diet containing 25% FM was used as a control (Diet A), and three other diets were formulated to contain 15% FM and supplemented with 0, 0.75, and 1.5% *S. limacinum* (Diet B, C, and D, respectively). The experiment was carried out in quadruplicates (30 shrimp per replicate, average weight 1.01 ± 0.01 g), and the shrimps were fed the test diets to apparent satiation three times daily for 8 weeks. Shrimp fed diet B and D showed lower weight gain than those fed diet A. Supplementation of 0.75% *S. limacinum* enhanced expression of antioxidative genes (superoxide dismutase and catalase) and immune-response-related genes in hepatopancreas but could not affect the gene expression of immune deficiency in hepatopancreas and Tube in the intestine. A low FM diet induced endoplasmic reticulum swelling of the intestinal epithelial cells, which was alleviated by *S. limacinum* supplementation. Ultra-performance liquid chromatography coupled with quadrupole time of flight mass spectrometry was employed to analyze the changes of hemolymph metabolomics, 49 significantly different metabolites were identified, and lysoPCs, deoxyinosine, inosine, and highly unsaturated fatty acids were lower in fish fed with low FM diets. Intestinal microbial diversity was lower in shrimp fed Diet B than those fed the control diet. Dietary supplementation of 0.75% *S. limacinum* increased intestinal microbial diversity of shrimp and decreased the ratio of pathogenic bacterium (*Thalassotalea* and *Tenacibaculum*). These results indicated that supplementing *S. limacinum* into a low FM diet improves the growth performance, immune response, and intestinal health of *P. monodon*. The optimum inclusion level of seems to be 0.75% of diet.

## Introduction

Fish-meal (FM) is the most important dietary protein source for shrimp and carnivorous fish (Bendiksen et al., 2011; Olsen and Hasan, 2012; Xie et al., 2014). Due to the global expansion of aquaculture, the limited production of FM has become a restrictive factor for the speedy and sustainable development of aquaculture.

*Penaeus monodon* has been one of the most abundantly cultured shrimp species in the world, and it has an annual yield of over 700,000 tons (FAO, 2018). Despite this, research into FM replacement in *P. monodon* is much less than those in *Litopenaeus vannamei* (Amaya et al., 2007; Ye et al., 2011; Yue et al., 2012; Kuhn et al., 2016). In China, the country with the biggest shrimp production, FM levels in commercial shrimp feed are still very high (>15%) (Rahimnejad et al., 2018). Our previous research showed that supplementation of essential nutrients in low FM diets can prevent suppression of the immune response (Wang et al., 2009; Xie et al., 2016, 2018; Xie S. et al., 2019).

*Schizochytrium limacinum* is a microalgae, Xie J. et al. (2019) has reported that dietary supplementation of *S. limacinum* could improve the growth performance of golden pompano. Since *S. limacinum* is rich in docosahexaenoic acid (DHA), it has been tested as an alternative to fish oil in fish and shrimp feed (García-Ortega et al., 2016; Wang et al., 2017a, b). Docosahexaenoic acid has been shown to reduce oxidative stress, inhibite inflammation, and modulate apoptosis and autophagy in many studies (Vincentini et al., 2011; Kim et al., 2014; Jeromson et al., 2015). Due to the high content of DHA, *S. limacinum* is also reported to enhance the immune function of white shrimp and golden pompano (Non-wachai et al., 2010; Xie J. et al., 2019). To date, there is no report on the application of *S. limacinum* for *P. monodon*. Accordingly, this study was designed to evaluate whether supplementation of *S. limacinum* in a low FM diet could improve immune function and intestinal health of *P. monodon*.

Recently, increasing attention is being paid to the relationship between diet, gut microbiota, and host health (Shapiro et al., 2014; Rooks and Garrett, 2016). In this study, ultra-performance liquid chromatography coupled with quadrupole time of flight mass spectrometry (UPLC-QTOF-MS) was employed to evaluate the effects of dietary supplementation with *S. limacinum* on hemolymph metabolites profile of shrimp, and 16S rRNA was employed to explore the changes of intestinal microbiota induced by *S. limacinum*. UPLC-QTOF-MS and 16S rRNA were combined to find out the potential mechanism through which *S. limacinum* enhances the immune response in shrimp.

## Materials and Methods

### Ethics Statement

This study was carried out in accordance with the recommendations of Care and Use of Laboratory Animals in China, Animal Ethical and Welfare Committee of China Experimental Animal Society. The protocol was approved by the Animal Ethical and Welfare Committee of Sun Yat-sen University (Guangzhou, China).

### Diet Preparation

Four practical diets were formulated, a 25% FM diet was used as a control (diet A), and three other diets were prepared by replacing 10% of FM with soy protein concentrate. Low FM diets were supplemented with 0, 0.75, and 1.5% *S. limacinum* (diet B, C and D, respectively). *S. limacinum* was provided by Kingdomway (Xiamen, China), which contained 19.4% DHA. Formulation and proximate composition of the diets are presented in Table 1, and fatty acid compositions of the diets are showed in Supplementary Table 1. Crystalline amino acids were mixed and precoated with 1% carboxymethyl cellulose to prevent leaching loss. The extra supplemented vitamins and minerals were dissolved in 200 mL water and then mixed with the dry ingredients. The 1.2 mm diameter pellets were cold-extruded using a pelletizer (Institute of Chemical Engineering, South China University of Technology, Guangdong, China), heated in an electric oven at 90°C for 60 min, then air-dried to approximately 10% moisture, and stored at -20°C until used.

**TABLE 1:** Formulation and proximate composition of experimental diets (% dry matter).

**Ingredients**	**A**	**B**	**C**	**D**
Fish meal^a^	25.00	15.00	15.00	15.00
Soybean meal^a^	25.00	25.00	25.00	25.00
Peanut meal^a^	12.00	12.00	12.00	12.00
Soy protein concentrate^b^	0.00	9.00	9.00	9.00
Wheat flour	23.20	21.04	20.79	20.54
Beer yeast^a^	2.00	2.00	2.00	2.00
Shrimp meal^a^	2.00	2.00	2.00	2.00
Chicken meal^a^	3.00	3.00	3.00	3.00
Fish oil^a^	1.00	2.00	2.00	2.00
Soy oil	1.50	1.50	1.00	0.50
*Schizochytrium limacinum*^c^	0.00	0.00	0.75	1.50
Choline chloride (50%)	0.20	0.25	0.25	0.25
Soy lecithin^a^	1.00	1.00	1.00	1.00
Vitamin and mineral mixture^d^	2.00	2.00	2.00	2.00
Monocalcium phosphate	1.00	2.00	2.00	2.00
Vc	0.10	0.10	0.10	0.10
Met-Met^e^	0.00	0.20	0.20	0.20
L-lysine monohydrochloride^f^	0.00	0.25	0.25	0.25
Threonine^f^	0.00	0.05	0.05	0.05
Glycine^f^	0.00	0.25	0.25	0.25
Alanine^f^	0.00	0.15	0.15	0.15
GABA^f^	0.00	0.02	0.02	0.02
Taurine^f^	0.00	0.10	0.10	0.10
Ornithine^f^	0.00	0.02	0.02	0.02
Phytase^g^	0.00	0.04	0.04	0.04
Vitamin B12	0.00	0.001	0.001	0.001
Vitamin B6	0.00	0.001	0.001	0.001
ZnSO_4_⋅7H_2_O	0.00	0.02	0.02	0.02
FeSO_4_.7H_2_0	0.00	0.02	0.02	0.02
KI	0.00	0.0001	0.0001	0.0001
Folate	0.00	0.0001	0.0001	0.0001
Na_2_SeO_3_	0.00	0.00006	0.00006	0.00006
CMC	1.00	1.00	1.00	1.00
Proximate composition (%)				
Dry matter	90.16	89.35	89.19	90.23
Crude protein	41.35	42.22	42.34	42.04
Crude lipid	6.70	6.35	6.43	6.32

*^*a*^Supplied by Haida Feed Corporation, Guangzhou, China. ^*b*^Supplied by Wilmar, Beijing, China. ^*c*^Supplied by Kingdomway, Xiamen, China, protein, 17.5%; lipids, 48.6%; DHA, 19.4%; EPA, 5.8%; palmitic acid, 7.3%; others, 3%. ^*d*^Supplied by Ashare, Guangzhou, China. Composition of vitamin and mineral mixture (kg^–1^ of mixture): vitamin A, 250,000 IU; riboflavin, 750 mg; pyridoxine HCL, 500 mg; cyanocobalamin, 1 mg; thiamin, 500 mg; menadione, 250 mg; folic acid, 125 mg; biotin, 10 mg; a-tocopherol, 3,750 mg; myo-inositol, 2,500 mg; calcium pantothenate, 1,250 mg; nicotinic acid, 2,000 mg; vitamin D3, 45,000 IU; vitamin C, 7,000 mg, Zn, 4,000 mg; K, 22,500 mg; I, 200 mg; NaCl, 2.6 g; Cu, 500 mg; Co, 50 mg; FeSO_4_, 200 mg; Mg, 3,000 mg; Se, 10 mg. ^*e*^Supplied by Evonik, Beijing, China. ^*f*^Supplied by Aladdin Industrial Corporation, Shanghai, China. ^*g*^Supplied by Yiduoli Biotechnology Co., Ltd, Zhuhai, China.*

### Shrimp and Experimental Conditions

Juvenile *P. monodon* were obtained from the Shenzhen experimental base of South China Sea Fisheries Research Institute (Shenzhen, China). Prior to the experiment, shrimp were acclimated for 3 weeks and fed the commercial diet (25% FM). At the beginning of the experiment, 480 shrimp with average weight 1.01 ± 0.01 g were distributed randomly into 16 cylindrical fiberglass tanks (500 L), at a density of 30 shrimp in each tank. Each diet was randomly assigned to four tanks. All shrimp were fed to apparent satiation three times daily at 8:00, 13:00, and 18:00 for 8 weeks. Any uneaten feed was collected by siphoning and used to calculate feed intake.

During the experimental period, water temperatures ranged from 26 to 30°C, salinity was approximately 30‰, pH 7.7–8.0, ammonia nitrogen was lower than 0.05 mg L^–1^, and dissolved oxygen was higher than 6.5 mg L^–1^. Water quality parameters were measure by following the instructions of the detection kit (Sangpu, Beijing, China). Natural light-dark cycle was used during the trial.

### Sample Collection and Chemical Analyses

After the 8 weeks feeding trial, shrimp from each tank were weighed, counted, and sampled 24 h after the last feeding. Eight shrimp per tank were used to collect hemolymph samples, and hemolymph were taken from the pericardial cavity using 1-ml syringe and then centrifuged (8,000 rpm) at 4°C for 10 min. Supernatant was pooled and stored at −80°C until analysis of the enzymatic activity and metabolomics. Hepatopancreas and intestine from two shrimp per tank were immediately flash-frozen in liquid nitrogen and stored at −80°C prior to RNA extraction.

Moisture, crude protein, and crude lipid contents in the diets were determined using standard methods (AOAC, 1995). Moisture was determined by drying at 105°C until constant weight. Crude protein was measured by the Kjeldahl method after acid digestion using an Auto Kjeldahl System (1030-Auto-analyzer; Tecator, Hoganos, Sweden). Crude lipid was determined by the ether-extraction method using a Soxtec System HT (Soxtec System HT6; Tecator, Sweden). Fatty acid compositions in diets were measured by China National Analytical Center (Guangzhou, China). Lipids were extracted by a mixture of chloroform and methanol (2:1, v/v), the lipid phase was evaporated, and fatty acids were then saponificated by potassium hydrate. Fatty acids methyl esters were separated and quanitified by a gas chromatograph (GC 7820A, Agilent, United States) equipmented with a HP-88 column (30 cm × 0.25 mm).

### Hemolymph Biochemical Indices

Lipid-related metabolits and immune-related parameters in hemolymph were determined in this study. Total cholesterol (TC) contents were determined by cholesterol oxidase-peroxidase method (Allain et al., 1974). The contents of glutathione (GSH) were measured by its ability to react with DTNB (dithiobis-2-nitrobenzoic acid), and malondialdehyde (MDA) was measured by thiobarbituric acid method (Janero, 1990). The activities of superoxide dismutase (SOD) were determined by a hydroxylamine method (Masayasu and Hiroshi, 1979). All these parameters were determined following the instructions of the detection kit (Nanjing Jiancheng Bioengineering Institute, China), and they were measured by monitoring the absorbance changes in a microplate reader (TECAN infinite M200, Switzerland).

### Intestinal Histological Examination

Hematoxylin/eosin (H&E) staining and transmission eletron microscopy (TEM) were employed to evaluate the intestinal damage of shrimp. The same section of middle intestine from two shrimp per tank were fixed in Bouin’s solution for 12 h and then fixed in 70% ethanol. All fixed samples were dehydrated in a graded series of ethyl alcohol and embedded in paraffin. Sections (4 μm thickness) were stained with H&E and observed under a light microscope (Nikon Ni-U, Japan).

Another two intestinal samples per tank were used for TEM examination. As described by Zhang et al. (2012), samples were firstly fixed in 2.5% glutaraldehyde solution and then fixed in 1% OsO4 for 1 h, dehydrated in a graded series of ethyl alcohol, and embedded with resin. Resin blocks were sectioned using a diamond knife. Ultrathin sections (∼90 nm) from each sample were cut and placed on copper grids. Sections were stained with saturated uranyl acetate solution for 30 min, rinsed with distilled water and poststained with lead citrate for 30 min. Ultrathin sections were then screened with a TEM (JEOL JEM-1400, Japan) at 150 kV.

### Quantitative Real-Time PCR Analysis

Total RNA from hepatopancreas and intestine was extracted with RNAiso Plus reagent according to the manufacturer’s instructions (TaKaRa, Japan). Agarose gel electrophoresis and spectrophotometric analysis (A260:A280 nm) were used to assess RNA quality and concentration. cDNA was synthesized using a PrimeScriptTM RT reagent kit with gDNA Eraser (Takara, Japan), according to the manufacturer’s instructions.

Real-time PCR for the target genes were performed using a SYBR^®^ Premix Ex Taq^TM^ II (Takara, Japan) and quantified on the LightCycler 480 (Roche Applied Science, Basel Switzerland). As described by manufacturer’s introduction, 400 nM of forward- and reverse-specific primers, 10 ng of cDNA template, and nuclease-free water to a final volume of 10 μL were used. Then we used the following program: denaturation step at 95°C for 1 min, 40 amplification cycles of 5 s denaturation at 95°C, 15 s annealing at 60°C, 20 s extension at 72°C, a melt-curve analysis, and, finally, cooling to 4°C. The efficiency of primers for each gene was previously evaluated by serial dilutions to ensure that it was close to 100% (Supplementary Table 2).

Normalization of real-time qPCR data was done by geometric averaging of multiple internal reference genes (elongation factor 1α and β-actin) (Vandesompele et al., 2002). Three independent duplicates were conducted for each of the data.

### UPLC-Q/TOF-MS Analysis

In total, 48 shrimp hemolymph samples were analyzed by UPLC-QTOF-MS in this research to investigate the impact of dietary *S. limacinum* on the lipid profiles of shrimp with 12 shrimp for each group. Briefly, 400 μL acetonitrile/methanol (9:1) was added to 100 μL hemolymph, and the mixture was vortexed for 30 s. The mixture was centrifuged at 13,000 *g* for 10 min at 4°C, and 200 μL supernatant was collected for UPLC-QTOF-MS analysis. Equal amounts of all samples were pooled as a QC sample for LC-MS system conditioning and quality control.

Ultra-performance liquid chromatography coupled with quadrupole time of flight mass spectrometry was performed on an ACQUITY UPLC system (Waters, Manchester, United Kingdom) coupled with a G2-Si HDMS QTOF mass spectrometer (Waters, Manchester, United Kingdom). Chromatographic separation was carried out at 40°C on an ACQUITY HSS T3 1-class column (2.1 mm × 100 mm, 1.8 μm, Waters). Mobile phase A consisted of 10 nM ammonium formate in water and 0.1% formic acid, and mobile phase B consisted of 10 nM ammonium formate in IPA/ACN (70/30) and 0.1% formic acid. The flow rate was 0.4 mL+/min. From the start to 0.5 min, B was held at 2%, linearly increased to 40% during the next 1 min, increased to 45% during the next 3.5 min, increased to 55% during the next 3 min, increased to 85% during the next 6 min, increased to 99% during the next 6 min, kept constant for 3 min, decreased to 2% in 0.1 min, and then kept constant for 2.9 min. Both positive and negative modes were performed and operated in continuum mode with an acquisition time of 0.3 s per scan. The scan range was set at 50–1500 *m/z*. The capillary was set at 2.5and 2 kV in positive ion mode and negative ion mode, respectively. Sampling cone voltages were set at 40 V in both modes. The desolvation temperature and gas flow were 350°C and 700 L/h, the source temperature was set at 120°C. Before analyzing the sample sequence, five QC samples were run, and, during the analysis of the sample sequence, one QC sample was run after every six injections.

The acquired mass data were imported to Waters Masslynx v4.1 software for peak detection and alignment. All of the data were normalized to the summed total ion intensity per chromatogram. We detected 4025 and 3233 features in positive and negative modes, respectively. All the features with CVs >30% were removed. The data were imported to SIMCA-P (Version 13.0, Umetrics, Sweden), where multivariate analysis were performe, mainly included principal component analysis (PCA) and orthogonal partial least squares discriminant analysis (OPLS-DA). The quality of the models is described by the *R*_2_ and *Q*_2_ values. *R*_2_ is defined as the proportion of variance in the data explained by the models and indicates goodness of fit. *Q*_2_ is defined as the proportion of variance in the data predicted by the model and indicates predictability, calculated by a cross-validation procedure. A default seven-round cross-validation in SIMCA-P was performed throughout to determine the optimal number of principal components and to avoid model overfitting.

Significantly different metabolites were selected on the basis of the combination of a statistically significant threshold of variable influence on projection (VIP) values obtained from the OPLS-DA model, *P* values from one way ANOVA analysis on the normalized peak areas, and the maximum fold change, where metabolites with VIP values larger than 1.0, *P* values less than 0.05, and fold change larger than 1.5 were included, respectively.

Metabolite identification was performed using Progenesis QI (Waters, Non-linear Dynamics, Newcastle, United Kingdom). LipidMaps, Chemspider and Human Metabolome databased (HMDB) were used for MS1 identification, and theoretical fragments were used for MS/MS identification. A score (total 60) of identified metabolites obtained from QI larger than 40 was regarded as acceptable, and anything else was regarded as uncertain.

Heatmap and cluster analyses were conducted using the pheatmap package based on R software (version 3.7.2).

### 16S rRNA Analysis

Total bacterial DNA in the intestine was extracted with a PowerFecal^®^ DNA Isolation kit. DNA samples were amplificated (Sutton et al., 2013) and then quantified using Quant-iT^TM^ dsDNA HS Reagent. High-throughput sequencing analysis of bacterial rRNA was performed by Illumina HiSeq 2500 at Biomarker Tchnologies Corporation (Beijing, China). The splicing and filtering of the raw data were performed using FLASH v1.2.7 and Trimmornatic v0.33, and cleaning of chimera were performed using UCHIME v4.2. OTUs were obtained by QIIME (version 1.8.0), Alpha diversity was evaluated by Mothur (version v.1.30). We used binary jaccard to estimate β diversity (QIIME).

### Calculations and Statistical Analysis

The parameters were calculated as follows:

Percentage weight gain (WG, %) = 100 × (final body weight - initial body weight)/initial body weight

Feed efficiency (FE) = (final body weight - initial body weight)/feed consumed

Survival (%) = 100 × (final amount of shrimp)/(initial amount of shrimp)

The results are presented as the means ± SEM (standard error of the mean). All the data were statistically analyzed by SPSS 19.0 (SPSS, Chicago, IL, United States). The data were first tested for homogeneity; if the data had similar variances, then a one-way ANOVA was used to test the main effect of dietary treatment. When there were significant differences (*P* < 0.05), the group means were further compared using Duncan’s multiple range test. When data did not have a homogeneous viaration, the non-parameter Kruskal–Wallis test was applied and followed by all pairwise multiple comparisons if the results of Kruskal–Wallis test shown significant difference (*P* < 0.05).

## Results

### Growth Performance

**FIGURE 1:**
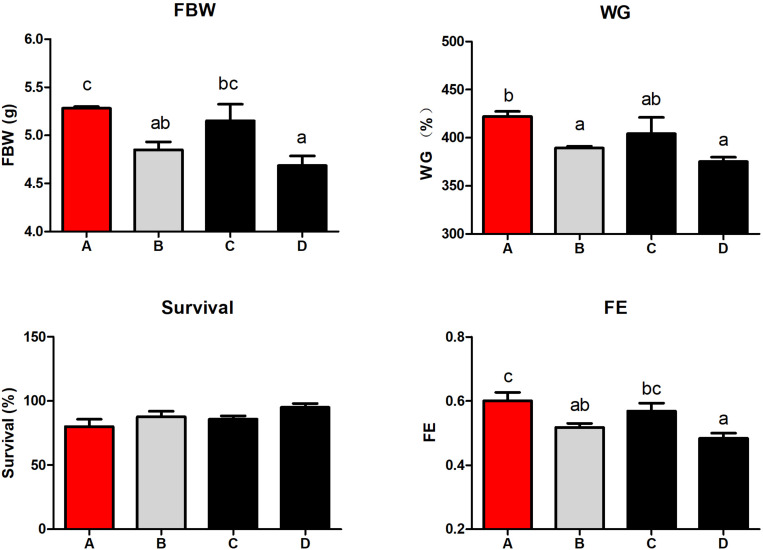
The effect of different diets on growth performance and feed utilization of shrimp. Values are means, with standard error represented by vertical bars. Mean values with different letters are significantly different (*P* < 0.05). Diet A, 25% fish meal; diet B, 15% fish meal; diet C, 15% fish meal + 0.75% *Schizochytrium Limacinum*; diet D, 15% fish meal + 1.5% *Schizochytrium Limacinum.* FBW, final body weight; WG, percentage weight gain; FE, feed efficiency.

The final body weight (FBW), WG, and FE were higher in shrimp fed diet A than those fed diet B and D (*P* < 0.05; Figure 1), and there were no differences between group A and C. Survival was similar between all the groups (*P* > 0.05).

### Biochemical Parameters of Hemolymph and Hepatopancreas

**FIGURE 2:**
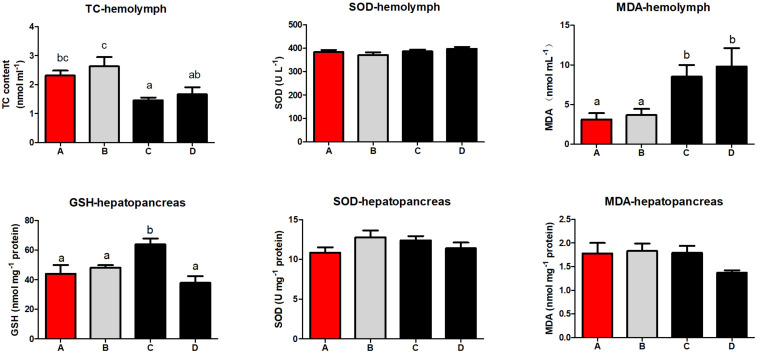
Effect of different diets on hemolymph and hepatopancreas physiologique index of *Penaeus monodon*. TC, total cholesterol; SOD, superoxide dismutase; MDA, malonaldehyde; GSH, glutathione.

Hemolymph TC content in shrimp fed diet B was higher than those fed diets containing *S. limacinum* (*P* < 0.05; Figure 2). *S. limacinum* supplementation significantly increased hemolymph MDA content of shrimp (*P* < 0.05). The GSH content in hepatopancreas was higher in group C than the other groups (*P* < 0.05). The SOD activities in hemolymph and hepatopancreas showed no significant differences between the four groups, the MDA contents in hepatopancreas showed a similar trend (*P* > 0.05).

### Hepatopancreatic Immune Response

**FIGURE 3:**
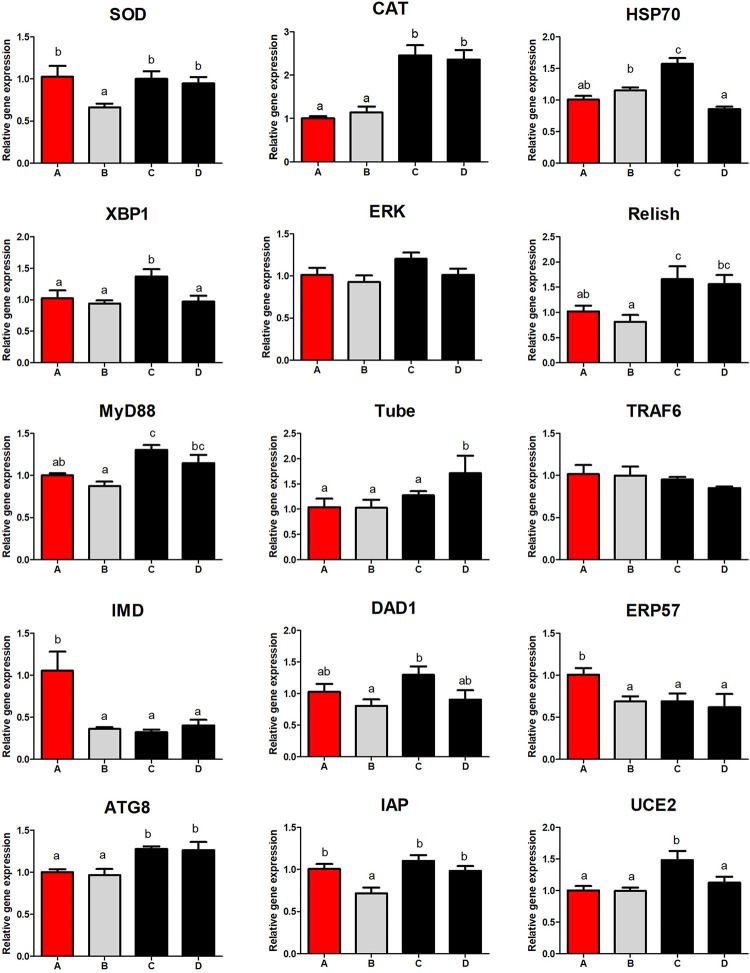
Effect of different diets on hepatopancreas immune response of *Penaeus monodon*. SOD, superoxide dismutase; IAP, inhibitor of apoptosis proteins; IMD, immune deficiency; ERP57, endoplasmic reticulum protein57; CAT, catalase; MyD88, myeloid differentiation primary response gene 88; ATG8, autophagy-related protein 8; ERK, extracellular signal-regulated kinase; HSP70, heat shock protein 70; XBP1, X-box binding protein 1; DAD1, defender against cell death 1; UCE2, ubiquitin conjugated enzyme 2.

Low FM diet suppressed mRNA expression of immune deficiency (IMD) and endoplasmic reticulum protein57 (ERP57) in the hepatopancreas (*P* < 0.05), and supplementation of *S. limacinum* had no effect on their expression (Figure 3). However, *S. limacinum* supplementation alleviated the low expression of SOD and inhibitor of apoptosis proteins (IAP) induced by a low FM diet (*P* < 0.05). Shrimp fed diets containing *S. limacinum* obtained higher catalase (CAT), myeloid differentiation primary response gene 88 (MyD88), and autophagy-related protein 8 (ATG8) mRNA expression compared to those fed other diets (*P* < 0.05). Shrimp fed diet C obtained the highest mRNA expression of heat shock protein 70 (HSP70), Relish, X-box binding protein 1 (XBP1), defender against cell death 1 (DAD1), and ubiquitin conjugated enzyme 2 (UCE2) between all the groups. The mRNA level of Tube in group D was higher than that of other groups.

### Intestinal Immune Response

**FIGURE 4:**
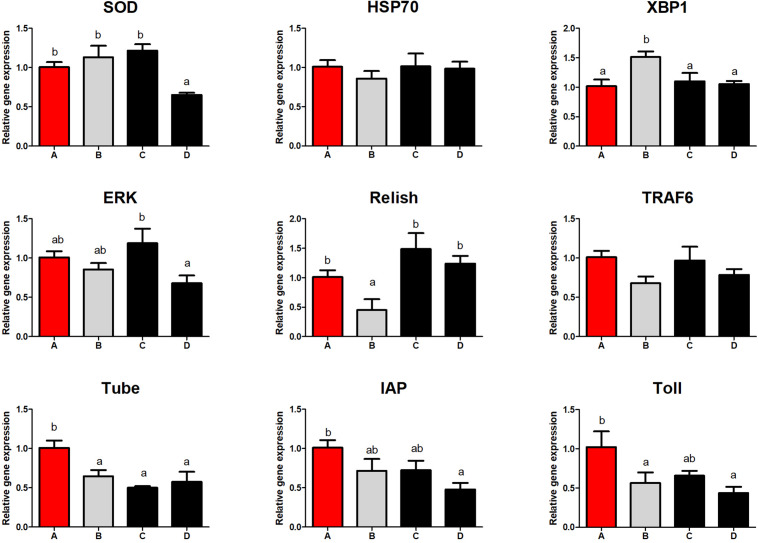
Effect of different diets on intestinal immune response of *Penaeus monodon*. SOD, superoxide dismutase; IAP, inhibitor of apoptosis proteins; ERK, extracellular signal-regulated kinase; HSP70, heat shock protein 70; XBP1, X-box binding protein 1; TRAF6, tumor necrosis factor receptor associated factor 6.

Shrimp fed diet B obtained lower Relish and higher XBP1 expression than those fed other diets (*P* < 0.05; Figure 4). Superoxide dismutase, extracellular signal-regulated kinase (ERK), Toll, and IAP mRNA levels in the intestine were lowest in shrimp fed diet D (*P* < 0.05). Low dietary FM downregulated the mRNA expression of Tube (*P* < 0.05). There were no differences in the expression of HSP70 and tumor necrosis factor receptor associated factor 6 (TRAF6) among all the treatment groups (*P* > 0.05).

### Intestinal Histology

**FIGURE 5:**
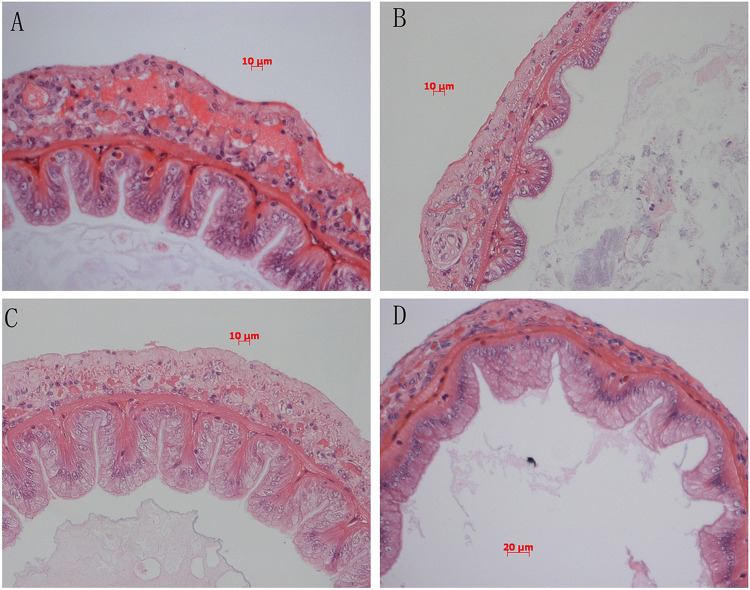
Effect of different diets on intestinal histology of *Penaeus monodon*. **(A–D)** Represent the intestinal histology of different groups.

**FIGURE 6:**
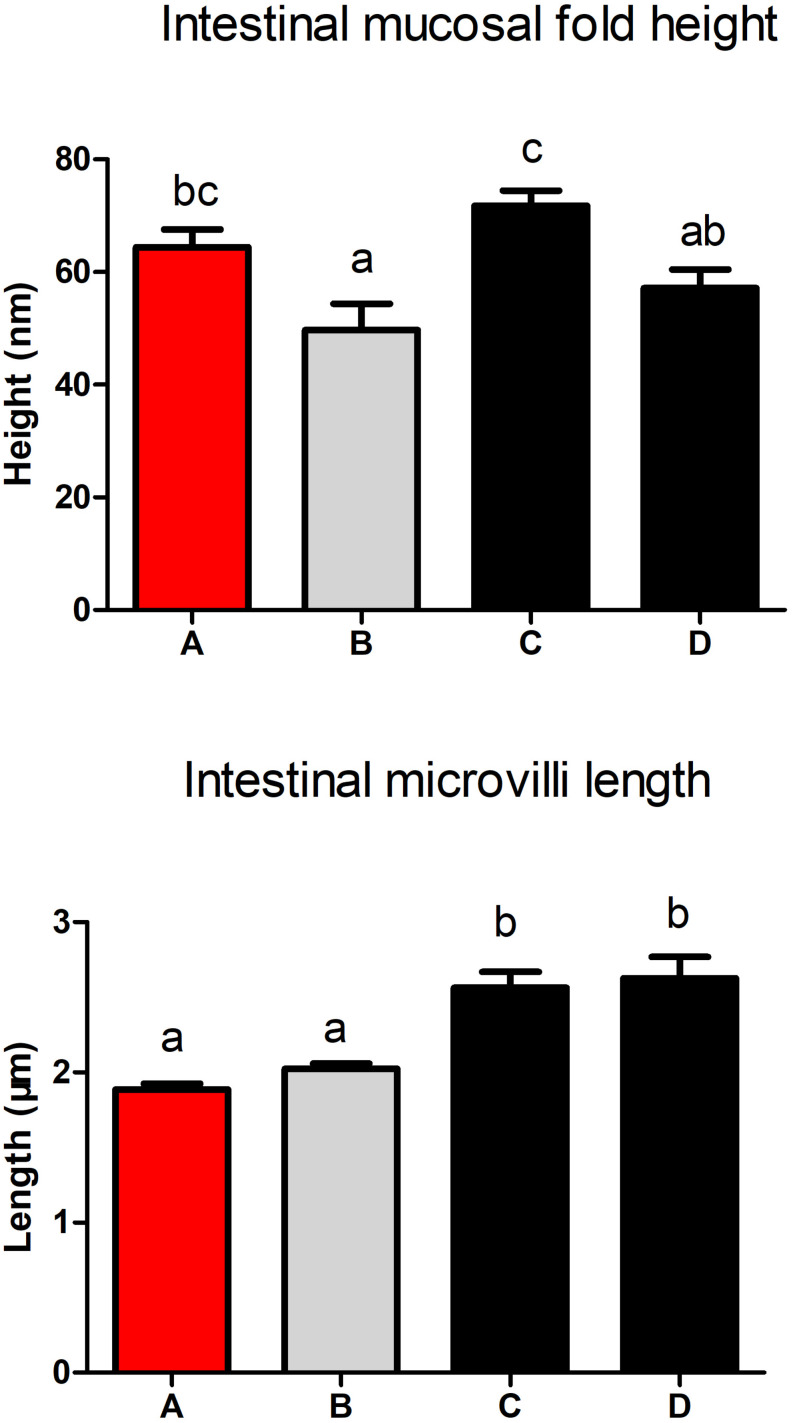
Effect of different diets on intestinal mucosal fold and microvilli of *Penaeus monodon*.

**FIGURE 7:**
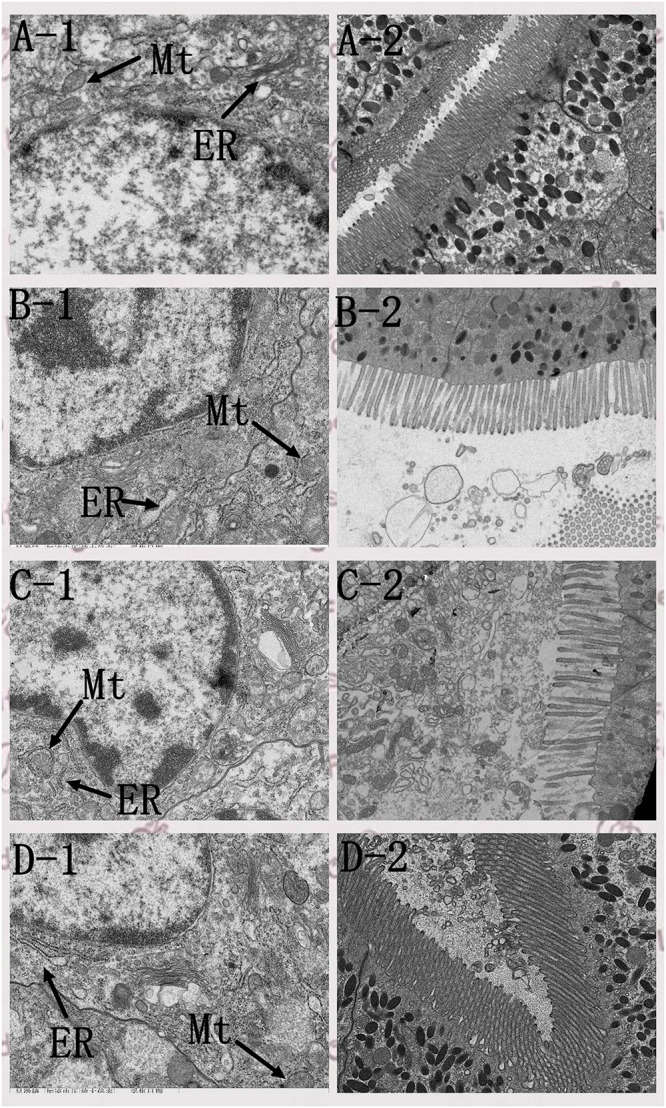
Effect of different diets on intestinal transmission electron microscope ultramicrotomy of *Penaeus monodon*. Mt, mitochondria; ER, endoplasmic reticulum. **(A–D)** Represent the intestinal histology of different groups. The number 1 represent the visual field of cell nucleus and organelle, the number 2 represent the visual field of microvilli.

Intestinal H&E staining showed that intestinal morphology were adversely affected by diet B (Figure 5), the intestinal fold height was lower in shrimp fed diet B compared with those fed diets A and C (Figure 6). TEM analysis also demonstrated that the intestine suffered more serious damage in shrimp fed diet B (Figure 7), especially the endoplasmic reticulum (ER), which showed obvious swelling in group B. The ER back to normal after dietary supplementation with *S. limacinum*, and *S. limacinum* supplementation increased the microvilli length of shrimp (*P* < 0.05).

### Multivariate Analysis and Biomarker Identification

**FIGURE 8:**
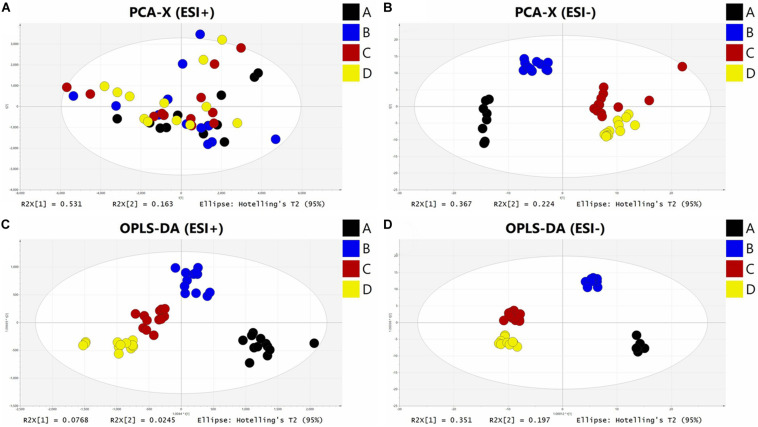
The PCA and OPLS-DA analysis of hemolymph samples in ESI + mode **(A,C)** and ESI- mode **(B,D)**.

In this study, a non-targeted metabolomics strategy was applied. PCA was applied to evaluate the separation between the four groups, Figures 8A,B show a clear cluster in ESI+ (*R*_2_*X* = 0.982, *Q*_2_ = 0.911) and ESI- (*R*_2_*X* = 0.775, *Q*_2_ = 0.654) modes. A supervised multivariate data analysis method, OPLS-DA, was established for testing differences between features that clearly distinguished the different groups in ESI+ (*R*_2_*X* = 0.906, *R*_2_*Y* = 0.62, *Q*_2_ = 0.497) and ESI- (*R*_2_*X* = 0.791, *R*_2_*Y* = 0.97, *Q*_2_ = 0.923) modes (Figures 8C,D), showed a goodness of fit (*R*_2_) and predictive ability (*Q*_2_) in this model.

Features with a VIP value larger than 1.0, *P* value less than 0.05 and fold change larger than 1.2 were regarded as significantly different features. In total, 218 significant features were found to satisfy the criterion (119 in ESI+ and 99 in ESI-). Among them, 41 in ESI+ and 54 in ESI-were identified, 20 in ESI+ and 39 in ESI- were regarded as reliable. Therefore, 59 significantly different metabolites were identified in this experiment and are presented in Table 2.

**TABLE 2:** The significantly different metabolites identified in hemolymph.

**Compound**	**Categories**	**Ions**	**m/z**	**Retention time (min)**	**Adducts**	**Formula**	**Score**	**Anova (p)**	**fold (B/A)**	**fold (C/A)**	**fold (D/A)**
Docosahexaenoic acid	lipid acids	−	327.2318846	12.4498	M - H	C_22_H_32_O_2_	57.7	0.005	0.62	0.61	0.71
Eicosapentaenoic acid			301.216419	11.87605	M - H	C_20_H_30_O_2_	46.9	0.011	0.91	0.85	0.78
Arachidonic acid		−	303.2316627	12.60691667	M - H	C_20_H_32_O_2_	57.7	0.018	0.72	0.71	0.69
LysoPE(22:6)	lysophosphatides	+	508.2809779	16.9678	M + H - H2O	C_27_H_44_NO_7_P	44.4	0.011	1.02	1.10	1.25
LysoPC(18:0)		−	568.360502	11.90748333	M + FA - H	C_26_H_54_NO_7_P	56.8	0.000	0.55	0.62	0.45
LysoPC(16:0)		−	540.3294645	10.48526667	M + FA - H	C_24_H_50_NO_7_P	50.5	0.000	0.54	0.55	0.52
LysoPC(18:1)		−	566.3450064	10.83093333	M + FA - H	C_26_H_52_NO_7_P	55.7	0.000	0.50	0.50	0.39
PE(20:5/20:5)	high unsaturated degree (≥ 5)	+	806.470794	14.80423333	M + Na	C_45_H_70_NO_8_P	41.5	0.000	1.01	0.90	0.80
PC(18:3/20:5)		−	846.5265432	14.42491667	M + FA - H	C_46_H_76_NO_8_P	52.7	0.000	0.69	0.58	0.60
PE(20:5/18:2)		−	760.4903205	14.98008333	M - H	C_43_H_72_NO_8_P	39.7	0.000	1.03	0.75	0.66
PC(22:6/16:1)		+	826.5360416	14.63665	M + H	C_46_H_78_NO_8_P	46.3	0.007	1.04	0.92	0.83
PE(P-16:0/22:6)		−	746.5112251	16.20295	M - H	C_43_H_74_NO_7_P	45.9	0.000	1.36	1.39	1.59
PE(22:6/16:0)		−	762.5053731	15.71241667	M - H	C_43_H_74_NO_8_P	43.6	0.000	1.93	1.85	1.87
PS(22:6/15:0)		+	794.4999741	16.5907	M + H	C_43_H_72_NO_10_P	45.6	0.011	1.27	1.24	1.48
PE(22:6/P - 18:0)		−	774.5425302	17.27963333	M - H	C_45_H_78_NO_7_P	52.8	0.003	1.19	1.33	1.55
PE(20:1/20:4)		−	838.5583828	15.57585	M + FA - H	C_45_H_80_NO_8_P	42.5	0.001	1.50	1.26	1.34
PC(14:0/22:5)		−	760.5252399	16.70575	M - H2O - H	C_44_H_78_NO_8_P	51	0.000	1.26	1.41	1.75
PC(20:5/16:0)		+	802.535078	15.01136667	M + H	C_44_H_78_NO_8_P	52.2	0.048	1.13	1.07	1.06
PC(18:4/16:1)		+	774.503595	14.39571667	M + H	C_42_H_74_NO_8_P	50.5	0.021	1.22	1.20	1.35
PE(16:0/20:5)		−	736.4906385	15.40786667	M - H	C_41_H_72_NO_8_P	45.7	0.000	1.43	1.33	1.21
PE(22:5/P-18:0)		−	776.5566727	17.45771667	M - H	C_45_H_80_NO_7_P	52.4	0.014	1.25	1.22	1.50
PC(P-16:0/18:4)	moderate unsaturated degree (2–5)	−	782.5316103	14.95913333	M + FA - H	C_42_H_76_NO_7_P	41.2	0.000	0.76	0.56	0.54
PS(20:2/17:2)		+	820.5085127	14.84613333	M + Na	C_43_H_76_NO_10_P	48.1	0.038	0.91	0.86	0.91
PE(16:0/20:4)		−	720.4950452	15.85945	M - H2O - H	C_41_H_74_NO_8_P	57.2	0.000	1.37	1.38	1.55
PC(18:2/16:1)		−	800.5436344	15.13491667	M + FA - H	C_42_H_78_NO_8_P	55.2	0.000	0.75	0.54	0.61
PC(18:3/16:1)		−	798.5258002	14.72868333	M + FA - H	C_42_H_76_NO_8_P	51.6	0.000	0.76	0.50	0.55
PC(17:1/18:2)		−	768.5519172	15.89088333	M - H	C_43_H_80_NO_8_P	50	0.002	0.94	0.81	0.77
PS(17:1/22:2)		−	808.5465342	15.57585	M - H2O - H	C_45_H_82_NO_10_P	46.7	0.044	0.82	0.77	0.76
PS(18:3/20:0)		+	836.5432704	15.77603333	M + Na	C_44_H_80_NO_10_P	45	0.041	1.08	1.15	1.24
PC(18:2/22:1)		−	884.6346678	17.98145	M + FA - H	C_48_H_90_NO_8_P	52.3	0.000	1.88	1.93	2.09
PS(18:1/20:1)		+	816.5770341	16.05648333	M + H	C_44_H_82_NO_10_P	49.6	0.000	0.94	0.88	0.87
CDP-DG(18:2/18:0)		−	986.5324802	15.31338333	M - H2O - H	C_48_H_85_N_3_O_15_P_2_	39.5	0.000	1.29	1.41	1.39
PS(20:2/21:0)		−	838.593254	16.67431667	M - H2O - H	C_47_H_88_NO_10_P	48.7	0.000	0.71	0.70	0.64
PE(16:1/20:1)		−	742.5355571	17.10156667	M - H	C_41_H_78_NO_8_P	46.2	0.004	1.48	1.62	1.47
PS(16:1/24:1)		+	844.6045968	17.1435	M + H	C_46_H_86_NO_10_P	41.2	0.000	0.79	0.75	0.73
PC(16:1/16:1)		+	752.5194671	14.84613333	M + H	C_40_H_76_NO_8_P	52.8	0.025	0.86	0.78	0.85
PC(P-18:0/20:2)		+	836.5907768	14.5319	M + K	C_46_H_88_NO_7_P	40.7	0.000	1.38	3.37	7.05
PS(P-16:0/18:2)		−	788.5082455	15.82783333	M + FA - H	C_40_H_74_NO_9_P	51.3	0.000	1.49	1.49	1.58
SM(d18:1/16:0)	low unsaturated degree (0 or 1)	−	747.5630019	15.6389	M + FA - H	C_39_H_79_N_2_O_6_P	57.9	0.004	1.29	1.65	1.37
PC(P-18:1/14:0)		−	760.5464934	15.513	M + FA - H	C_40_H_78_NO_7_P	45.5	0.000	0.59	0.53	0.55
Trihexosylceramide (d18:1/12:0)		−	948.5887683	17.05966667	M - H2O - H	C_48_H_89_NO_18_	42.7	0.000	1.07	1.36	1.74
PC(P-18:0/16:1)		−	788.5777879	16.42291667	M + FA - H	C_42_H_82_NO_7_P	51.6	0.000	0.70	0.69	0.75
SM(d16:1/16:0)		+	675.5429773	14.64711667	M + H	C_37_H_75_N_2_O_6_P	39.5	0.025	1.25	1.34	1.30
LacCer(d14:1/16:0)		+	806.5649826	15.08468333	M + H	C_42_H_79_NO_13_	41.9	0.027	1.14	1.04	0.98
SM(d18:1/14:0)		−	719.5336692	14.85438333	M + FA - H	C_37_H_75_N_2_O_6_P	57.3	0.020	1.17	1.29	1.19
PS(17:0/20:0)		−	786.5615135	15.9223	M - H2O - H	C_43_H_84_NO_10_P	46	0.000	0.60	0.56	0.63
PS(16:0/21:0)		−	786.5618129	16.28675	M - H2O - H	C_43_H_84_NO_10_P	50.7	0.000	0.65	0.67	0.70
PC(16:0/16:0)		−	778.5580162	16.37055	M + FA - H	C_40_H_80_NO_8_P	53.8	0.007	1.10	1.30	0.99
PI-Cer(d18:0/20:0)		−	818.5875797	17.05966667	M - H2O - H	C_44_H_88_NO_11_P	51	0.043	1.08	1.18	1.26
LacCer(d14:0/18:0)		−	880.6030695	17.05966667	M + FA - H	C_44_H_85_NO_13_	51.7	0.000	1.07	1.36	1.78
PE(18:0/18:0)		+	786.5377163	15.0428	M + K	C_41_H_82_NO_8_P	40.5	0.000	0.72	0.68	0.70
PC(18:0/16:0)		+	784.5795931	15.63985	M + Na	C_42_H_84_NO_8_P	48.3	0.000	0.95	0.87	0.80
LacCer(d16:0/16:0)		+	858.5876461	16.7583	M + Na	C_44_H_85_NO_13_	45.8	0.000	1.10	1.36	1.71
PC(16:0/12:0)		+	678.5062372	14.59475	M + H	C_36_H_72_NO_8_P	40.7	0.010	1.30	1.20	1.16
2’-Deoxyinosine	others	−	251.0775001	1.483116667	M - H	C_10_H_12_N_4_O4	48.8	0.000	0.09	0.06	0.07
Inosine		−	267.0727504	1.483116667	M - H	C_10_H_12_N_4_O5	51.1	0.035	0.75	0.66	0.65
L-Tryptophan		−	203.0819896	1.8602	M - H	C_11_H_12_N_2_O_2_	57.5	0.000	0.70	0.44	0.53
6’-Hydroxysiphonaxanthin		+	835.5874912	16.73735	M + Na	C_53_H_80_O_6_	42.3	0.002	0.94	1.06	1.18
Cholic acid		+	817.5790619	16.3812	2M + H	C_24_H_40_O_5_	42.2	0.000	0.84	0.87	0.82

### Significantly Different Metabolites

**FIGURE 9:**
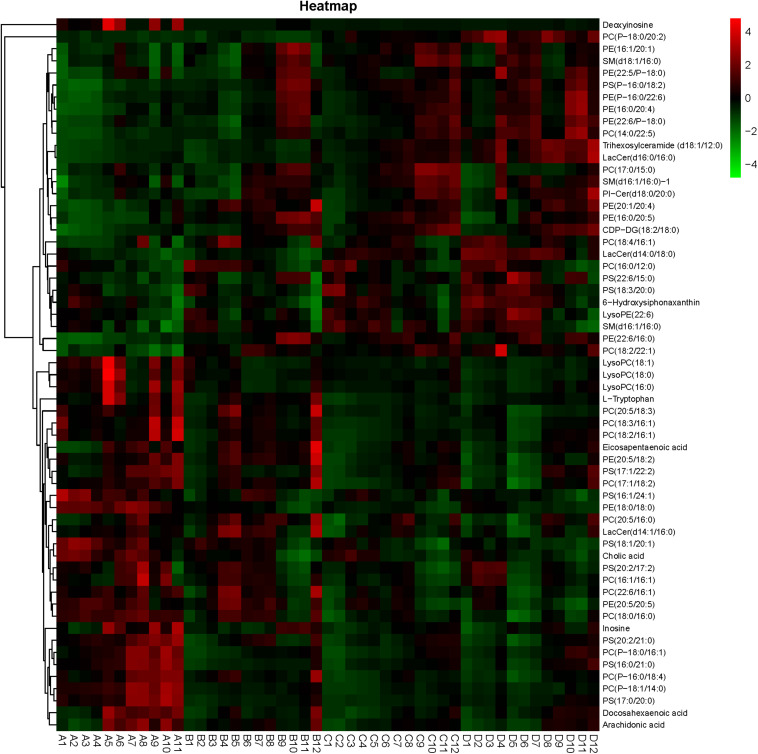
Heatmap of the differential metabolites among the different groups.

A heatmap based on significantly different metabolites showed clear differences between shrimp fed diet A and other diets (Figure 9). Most of these metabolites were lipids, especially phospholipids, including phosphatidylcholine (PC), phosphatidyl ethanolamine (PE), Phosphatidyl serine (PS), sphingolipid and fatty acids. Shrimp fed high FM showed higher levels of lowly unsaturated LysoPCs (LPC) and highly unsaturated fatty acids [HUFAs; DHA, eicosapentaenoic acid (EPA), and arachidonic acid (ARA)] in the hemolymph, than other groups (*P* < 0.05). Meanwhile, *S. limacinum* supplementation in low FM diets could not alleviate this situation. The proportion of 2′-deoxyinosine and inosine were lower in shrimp fed low FM diets than those fed a high FM diet, and a similar tendency was found in the proportions of L-tryptophan and cholic acid (*P* < 0.05). *S. limacinum* supplementation increased the content of lysoPE (LPE; 22:6) and 6′-hydroxysiphonaxanthin in hemolymph. There were no clear trends found in the effects of different diets on unsaturated degree of phospholipids.

### Intestinal Microbiota

A total of 3,027,778 high quality sequences were produced in this study, with an average of 189,236 sequences per sample. The coverage of each group exceeded 99%, which indicated that the 16S rRNA sequences identified in each group represented the majority of bacteria in the samples analyzed in this study. As shown in Table 3, the Shannon index was higher in shrimp fed a high FM diet compared to those fed diet B and D, which indicated the higher bacterial diversity in group A. There were no differences in OTUs numbers and community richness (Chao1 and ACE) between all the treatments. The analysis of PCoA showed that first two principal components explained 35.12% of the variations (Supplementary Figure 1).

**TABLE 3:** The Alpha diversity index of each groups.

	**A**	**B**	**C**	**D**
OTU	200 ± 9.08	196 ± 4.2	198.75 ± 5.62	195 ± 6.56
ACE	204.41 ± 7.55	200.16 ± 4.53	203.59 ± 4.88	203.77 ± 7.51
Chao1	206.3 ± 6.74	199.35 ± 4.34	206.23 ± 5.37	204.93 ± 7.58
Simpson	0.16 ± 0.03	0.29 ± 0.04	0.22 ± 0.04	0.31 ± 0.05
Shannon	2.72 ± 0.19b	2.04 ± 0.1a	2.24 ± 0.12*ab*	1.91 ± 0.1a

*^*a*,*b*^Mean values with unlike letters are significantly different (*P* < 0.05).*

The relative abundance of gut microbiota in each group at phylum level is shown in Supplementary Figure 2. The dominant phyla in the four groups were Proteobacteria, Bacteroidetes, Actinobacteria, Cyanobacteria, and Tenericutes. The relative abundance of Proteobacteria were exceeded 90% in all the groups. The relative abundance of Bacteroidetes, Actinobacteria, and Tenericutes were highest in shrimp fed diet A, while the relative abundance of Proteobacteria showed an opposite trend. The relative abundance of Cyanobacteria were highest in shrimp fed diet B and lowest in shrimp fed diet C.

**FIGURE 10:**
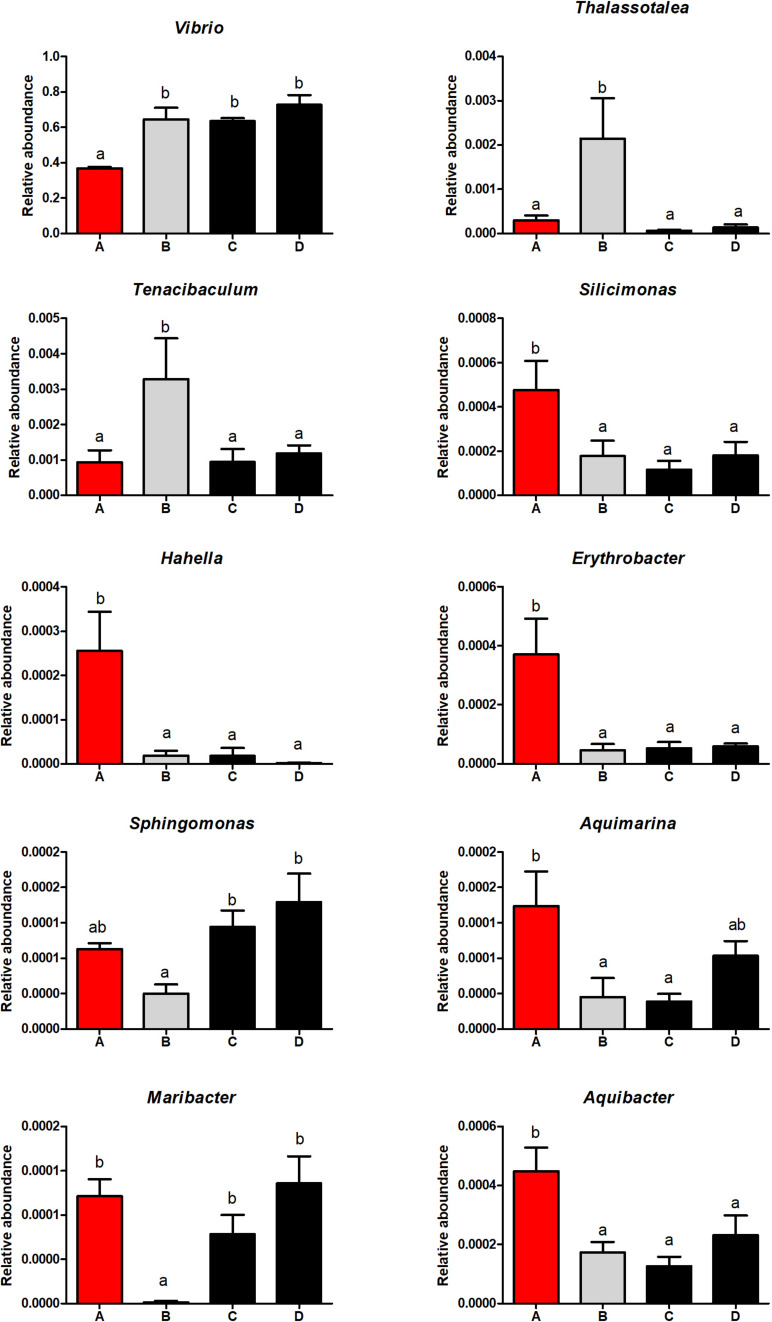
The significantly different intestinal microbiota in each group at the genus level.

The relative abundance of gut microbiota in each group at genus level is shown in Supplementary Figure 3. The dominant genera in four groups were *Vibrio*, *Pseudoalteromonas*, *Photobacterium*, *Halocynthiibacter*, and *Shimia*. The relative abundance of *Vibrio* was lower in shrimp fed the high FM diet, compared with those fed low FM diets (*P* < 0.05). The relative abundance of *Silicimonas*, *Hahella*, and *Erythrobacter* were higher in shrimp fed the high FM diet compared to those fed low FM diets (*P* < 0.05; Figure 10). The relative abundance of *Thalassotalea* and *Tenacibaculum* were higher in group B compared with that of the other groups (*P* < 0.05), while the relative abundance of *Maribacter* showed an opposite tendency. *S. limacinum* supplementation increased the relative abundance of *Sphingomonas* in the intestine.

## Discussion

The results of this study indicated that weight gain of *P. monodon* decreased when dietary FM decreased from 25 to 15%, even though fish oil, amino acids, and micro-nutrients were supplemented into the low FM diet to balance the nutrition profile. It was earlier reported that dietary FM in *P. monodon* could be reduced from 35 to 17.5% by soy protein concentrate without negative impact on growth performance (Paripatananont et al., 2001). The different results may due to the differences in environment and prawn strain. Interestingly, in this study suitable *S. limacinum* supplementation in low FM diet improved growth of shrimp. Previous research mainly focused on fish oil replacement with *S. limacinum* in feed of *L. vannamei* (Wang et al., 2017c; Perez-Velazquez et al., 2018; Araújo et al., 2019), and similar research also were carried out in *Epinephelus lanceolatus*, *Seriola rivoliana*, and *Sciaenops ocellatus* (García-Ortega et al., 2016; Kissinger et al., 2016; Perez-Velazquez et al., 2018). Xie J. et al. (2019) reported 3% *S. limacinum* could improve the growth of *Trachinotus ovatus*. This is the first study to reveal the direct growth promoting effect of *S. limacinum* in shrimp. The high content of DHA, EPA, and micronutrients such as carotenoid may contribute to the growth promoting effect of *S. limacinum* (Xie J. et al., 2019). Although 0.75% *S. limacinum* enhanced the growth performance of *P. monodon*, 1.5% *S. limacinum* did not produce a similar effect. Previous research also reported that high levels of *S. limacinum* impaired the growth of white shrimp and blunt snout bream (Wang et al., 2017b, 2020). High levels of *S. limacinum* may induce an imbalance of dietary fatty acids composition, and this consequently results in suppression of shrimp growth.

*Schizochytrium limacinum* supplementation showed a large impact on antioxidative ability and immune response of *P. monodon* in this study. Superoxide dismutase and CAT are two important antioxidative related genes (Lu et al., 2017), supplementation of *S. limacinum* in low FM diet upregulated their expression, also increased the andioxidant (GSH) content in hepatopancreas. Similar result has also been observed in *L. vannamei* (Non-wachai et al., 2010). The antioxidative role of *S. limacinum* may due to the high content of DHA, previous research indicated that DHA can decrease oxidative stress of animals by changing the composition of cell membrane (Ye et al., 2010). Docosahexaenoic acid is also able to inhibit inflammation, its derivatives resolvins, protectins and maresins are involved in inflammatory processes such as cytokines production and lymphocyte activation (Ye et al., 2010; Zhao et al., 2015). ER stress and NF-κB have been well documented to be the main signaling pathways which DHA modulates the immune response (Begum et al., 2012; Crnkovic et al., 2012). Compared with group B, suitable *S. limacinum* supplementation upregulated the gene expression of XBP1 in hepatopancrease and downregulated its expression in intestine. XBP1 is the marker of ER stress (Sano and Reed, 2013), the results indicated that *S. limacinum* supplementation may alleviate the ER stress in shrimp. Relish, MyD88 and Tube are involved in NF-κB signaling pathway (Qiu et al., 2014), the present gene expression data indicated that NF-κB signaling pathway was modulated by *S. limacinum*. This study also found that autophagy (ATG8) and apoptosis (IAP and UCE2) related genes expression were affected by *S. limacinum*, which may be a downstream response of the NF-κB signaling pathway (Calder, 2015). However, the low expression of two critical innate immunity gene (IMD and Toll) induced by low FM diet could not be alleviated by *S. limacinum* supplementation, which revealed the limited benifical effects of *S. limacinum*. These results indicated that suitable supplementation of *S. limacinum* in a low FM diet could enhance the antioxidative ability of shrimp and improve the immune response of shrimp mainly through modulating the NF-κB related signaling pathways.

The intestine is a very important digestive organ, and intestinal health is closely related to the immune response of shrimp (Rooks and Garrett, 2016). In this study, the intestinal fold height and microvilli length were lower in shrimp fed diet B, which indicated that intestinal histology and epithelial cell ultrastructure of shrimp were damaged by high level of SPC. The damage of the intestine would have affected the digestion and absorption of nutrients, which may be the main cause of the compromised growth performance and immune response. Suitable supplementation of *S. limacinum* could alleviate endoplasmic reticulum swelling, and this result was consistent with the expression of ER stress and NF-κB signaling pathway related genes. In addition, suitable supplementation of *S. limacinum* also increased microvilli length and improved the overall intestinal health of shrimp, similar results were found in golden pompano (Xie J. et al., 2019).

The present results obtained through UPLC-QTOF-MS indicated that shrimp fed on a low FM diet showed fewer HUFAs in hemolymph than those fed a high FM diet, and it was unfortunate that *S. limacinum* supplementation could not increase hemolymph HUFAs content. Since the HUFAs play a critical role in immune response, the low hemolymph HUFAs in low FM groups may need to be improved in future studies. LysoPCs and LPE are derived from PC and PE, respectively, and have been considered as inflammatory lipids involved in several immune-mediated disease (Rindlisbacher et al., 2018), they also participate in cell signaling, energy metabolism and storage and other biological processes (Bai et al., 2014). In some studies, LPC and LPE have been found to aggravate inflammation associated diseases (Xie et al., 2020), and they were found to exert their effects through different signaling pathways, such as NF-κB, PKC, and ERK (Zeng et al., 2017). However, in other studies, LPC and LPE were found to decrease in inflammatory condition (Bai et al., 2014; Zhou et al., 2014; Qian et al., 2017; Wang et al., 2017a). These inconsistencies results of various research have indicated that the relationship between inflammation and levels of LPC and LPE may depend on species and inflammation stage. In this study, decreased LPCs in low FM diet groups may be associated with inflammation, and *S. limacinum* supplementation had no benificial effect on these changes. Additionally, *S. limacinum* supplementation also had little influence on the low deoxyinosine, inosine, and cholic acid content induced by low FM diet. These results from UPLC-QTOF-MS revealed that *S. limacinum* supplementation enhancing the immune response may not through changing the metabolomics profile of shrimp.

There have been numerous studies that have evaluated the effects of different protein sources on the gut microbiota composition of aquatic animal (Zhou et al., 2018; Niu et al., 2019), and previous studies examining *P. monodon* evaluated the effect of marine invertebrate meals on gut microbiota (Simon et al., 2019). In this study, low dietary FM levels decreased the gut microbial diversity of shrimp and increased the ratio of harmful genera such as *Vibrio*, *Pseudoalteromonas*, and *Photobacterium*, which may be associated with the impaired intestinal health in shrimp. Similar to our results, a study in Atlantic salmon indicated that high content of soybean meal decreased the ratio of lactic acid, and increased the ratio of *Aeromonas* (Bakke-McKellep et al., 2007). Desai et al. (2012) also reported that plant ingredients changed the gut microbial composition, and the changes in gut microbiota may contribute to negative health outcomes. *Thalassotalea* and *Tenacibaculum* are two pathogenic bacterium found in aquatic animals (Costantini et al., 2017). *S. limacinum* supplementation in low FM diet significantly decreased their ratios in the intestine of shrimp, while *S. limacinum* had no effect on the ratios of *Vibrio*, *Pseudoalteromonas*, and *Photobacterium*. These results indicated that low dietary FM levels increased the ratio of harmful bacterial genera, *S. limacinum* supplementation could decrease some of these harmful bacterial genera (*Thalassotalea* and *Tenacibaculum*). Dietary supplementation of *S. limacinum* enhancing the immune response and intestinal health may through changing intestinal microbiota of shrimp.

In conclusion, a low FM diet decreased the weight gain of *P. monodon*, impaired the immune response, induced intestinal damage, and increased the ratio of harmful bacterial genera in shrimp. Dietary supplementation with 0.75% *S. limacinum* promoted growth performance of and improved the immune response and intestinal health of shrimp.

## Data Availability Statement

The sequencing data has been deposited into the Sequence Read Archive (accession: PRJNA632684).

## Ethics Statement

This study was carried out in accordance with the recommendations of Care and Use of Laboratory Animals in China, Animal Ethical and Welfare Committee of China Experimental Animal Society. The protocol was approved by the Animal Ethical and Welfare Committee of Sun Yat-sen University (Guangzhou, China).

## Author Contributions

SX, JN, YL, and LT designed the experiments. SX and DW carried out the experiments. SX wrote the manuscript. SX and BT revised the manuscript. All authors read and approved the final version of the manuscript.

## Conflict of Interest

The authors declare that the research was conducted in the absence of any commercial or financial relationships that could be construed as a potential conflict of interest.
