# Supplementary material for: Schizochytrium limacinum Supplementation in a Low Fish-Meal Diet Improved Immune Response and Intestinal Health of Juvenile Penaeus monodon
Source: Front Physiol. 2020 Jun 30;11:613. doi: 10.3389/fphys.2020.00613 (PMC7344155; doi:10.3389/fphys.2020.00613)
Supplement: Supplementary file 1 [file Data_Sheet_1.docx]

**Supplementary Table 1** The fatty acid composition of different diets (% total fatty acids).

| Fatty acid | A | B | C | D |
| --- | --- | --- | --- | --- |
| C12:0 | 0.056 | 0.12 | 0.065 | 0.067 |
| C14:0 | 2.8 | 3.8 | 3.5 | 3.5 |
| C14:1 | 0.05 | 0.085 | 0.067 | 0.067 |
| C15:0 | 0.29 | 0.32 | 0.37 | 0.41 |
| C16:0 | 16.2 | 16.7 | 16.8 | 17.2 |
| C16:1 | 3.8 | 4.4 | 4.3 | 4.3 |
| C17:0 | 0.48 | 0.52 | 0.56 | 0.6 |
| C17:1 | 0.57 | 0.72 | 0.69 | 0.69 |
| C18:0 | 5.2 | 5.4 | 5 | 4.8 |
| C18:1 | 22.8 | 20.9 | 19.5 | 18.4 |
| C18:2 | 26.7 | 23 | 22.4 | 20.7 |
| C18:3 | 3 | 2.7 | 2.6 | 2.4 |
| C20:0 | 0.79 | 0.8 | 0.77 | 0.74 |
| C20:1 | 0.86 | 1 | 0.97 | 0.93 |
| C18:4 | 0.57 | 0.88 | 0.86 | 0.87 |
| C20:2 | 0.12 | 0.12 | 0.11 | 0.11 |
| C20:3 | 0.65 | 0.61 | 0.6 | 0.59 |
| C20:4 | 1.7 | 1.8 | 1.9 | 1.9 |
| C22:1 | 0.074 | 0.082 | 0.085 | 0.082 |
| C20:5 | 5.1 | 6.9 | 6.7 | 6.6 |
| C24:0 | 0.3 | 0.28 | 0.28 | 0.27 |
| C22:4 | 0.28 | 0.3 | 0.3 | 0.29 |
| C24:1 | 0.24 | 0.33 | 0.31 | 0.31 |
| C22:5 | 1.4 | 1.4 | 2 | 2.6 |
| C22:6 | 4.8 | 5.1 | 6.8 | 8.4 |

**Supplementary Table 2** Real-time PCR primer sequences

| Target genes | Primer sequence (5' to 3') | Efficiency | Product size (bp) |
| --- | --- | --- | --- |
| EF1α F | GGTGCTGGACAAGCTGAAGGC | 99.30% | 168 |
| EF1α R | CGTTCCGGTGATCATGTTCTTGATG |  |  |
| 18S F | GCCGGCACGTTTACTTT | 97.89% | 121 |
| 18S R | ACCGAGGTCCTCTTCCATCA |  |  |
| IMD F | GGGTCCGTGTCCAGTGAT | 97.22% | 136 |
| IMD R | GCTGCGAGCGAGGGTTA |  |  |
| Tube F | CGAGTGCCAAGTATGCC | 98.18% | 208 |
| Tube R | CCCGTTCCAGGAGATTTA |  |  |
| ATG8 F | AGAGGAGGGCAGAGGGA | 102.37% | 229 |
| ATG8 R | GCTGAAGTTGGAGGAATAA |  |  |
| UCE2 F | CAGCTGATCCCCTCGTAGGA | 103.51% | 80 |
| UCE2 R | CCAGAGCCGTGCAATTCTATC |  |  |
| ERK F | ATGTTATCCAACCGTCCTC | 95.67% | 264 |
| ERK R | CAGAGCCTCCTCCACAG |  |  |
| ERP57 F | CTGGCTACCCTACCCTGAA | 106.35% | 113 |
| ERP57 R | GGCTGGTCCAACCTGTGA |  |  |
| IAP F | TACCCACTCTTCCAACCC | 101.98% | 271 |
| IAP R | TCTGCCATACTCATTATCTCAT |  |  |
| XBP1 F | TCCACATCATCAAGGACGAGC | 94.26% | 189 |
| XBP1 R | TCTGTGCGGCAACTCTATTCT |  |  |
| Relish F | TCTCCAGGTGAGCACTCAGTTG | 97.25% | / |
| Relish R | GCTGTAGCTGTTGCTGTTGTTGAG |  |  |
| SOD F | GCTGCTACAAAGAAGTTGGT | 94.69% | / |
| SOD R | GGACTGGAATGATCCAAAGC |  |  |
| MyD88 F | GTGCACCAGAGTCATTGTAG | 99.17% | / |
| MyD88 R | GGGAGTGGCAGAAACTTATC |  |  |
| CAT F | ACTCCCATTGCTGCTGTTCGT | 95.62% | 130 |
| CAT R | ATCCCAATTTCCTTCTTCTG |  |  |
| Toll F | CCAGGAGAATACATCCAAAACCA | 97.36% | / |
| Toll R | TGGCCCCACACACTCTCAAT |  |  |
| TRAF6 F | CGTGAGATCCTGCAGCTTAGTG | 102.16% | / |
| TRAF6 R | GCACATGACTGGCTGAAAAGAA |  |  |
| HSP 70 F | TCCTACGTCGCCTTCACAGAC | 106.48% | 189 |
| HSP 70 R | CTTTGGCTTTGTGCTCTCGTT |  |  |

EF1α, elongation factor 1α; IMD, immune deficiency; ATG8, autophagy-related protein 8; UCE2, ubiquitin conjugated enzyme 2; ERK, extracellular signal-regulated kinase; ERP57, endoplasmic reticulum protein57; IAP, inhibitor of apoptosis proteins; XBP1, X-box binding protein 1; SOD, superoxide dismutase; MyD88, myeloid differentiation primary response gene 88; CAT, catalase; TRAF6, TNF receptor associated factor 6; HSP 70, heat shock protein 70.


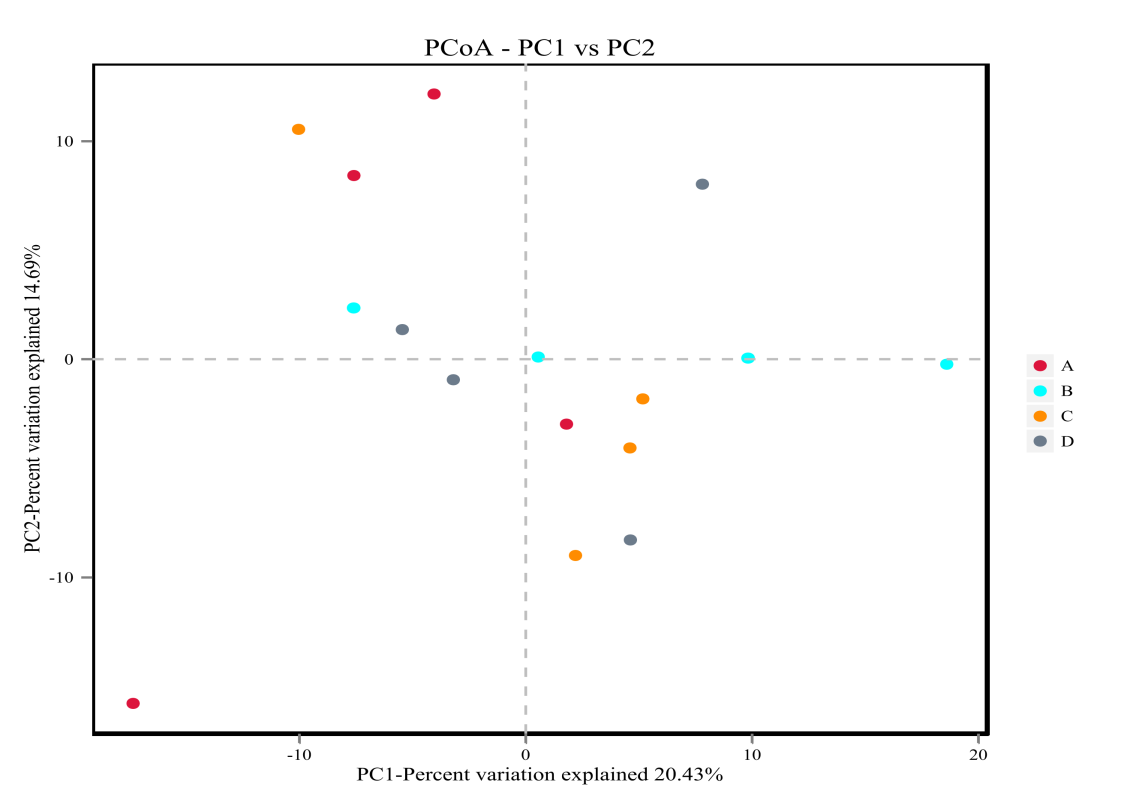
**Supplementary Fig. 1.** PCoA analysis of the intestinal microbiota samples .


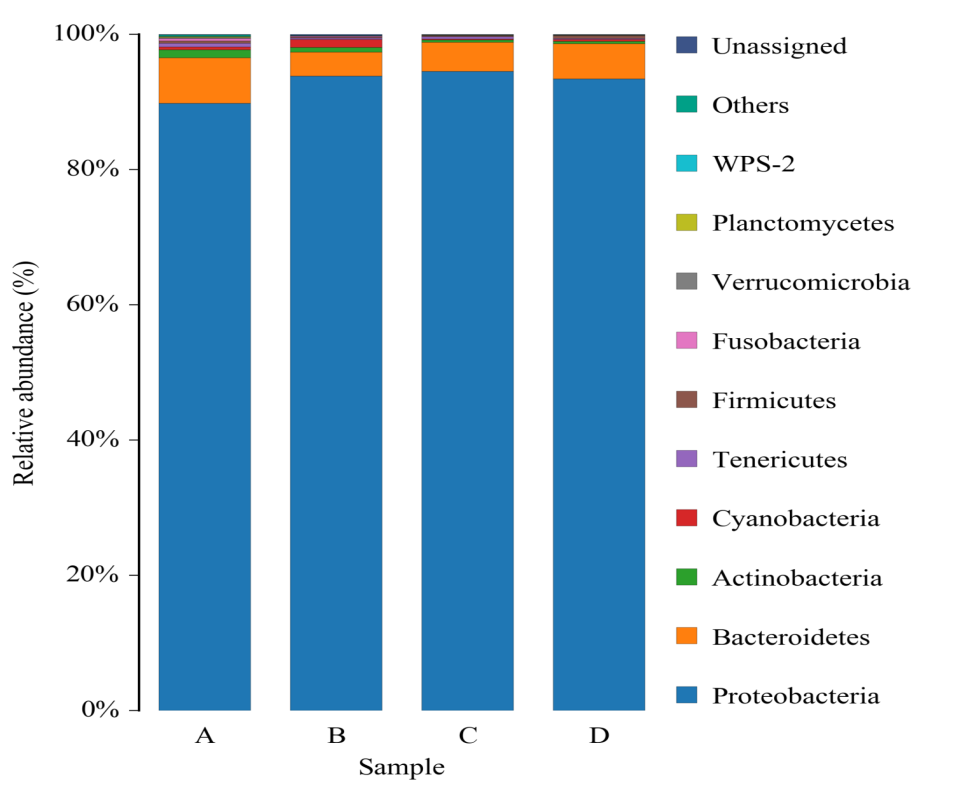
**Supplementary Fig. 2.** The relative abundance of intestinal microbiota in each group at the phylum level.

**Supplementary**
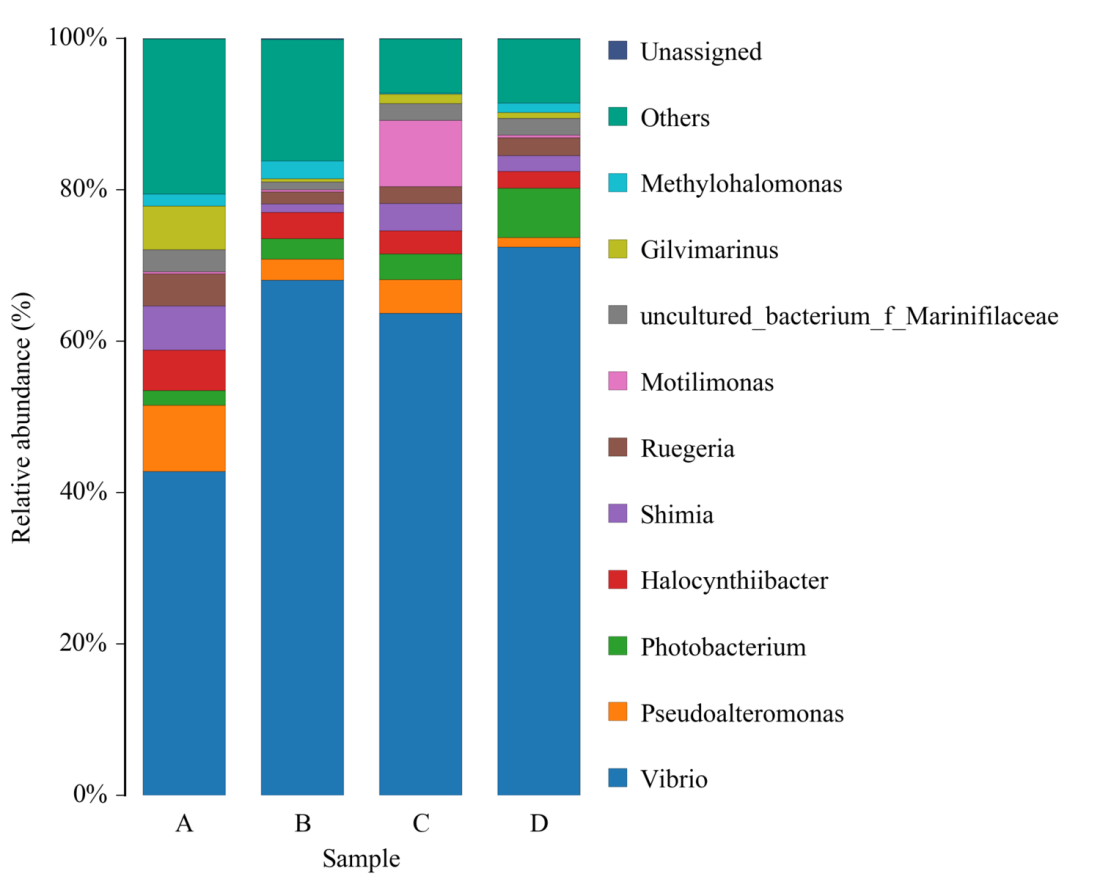
**Fig. 3.** The relative abundance of intestinal microbiota in each group at the genus level
